# The dynamics of neutralizing antibodies against SARS-CoV-2 in cats naturally exposed to virus reveals an increase in antibody activity after re-infection

**DOI:** 10.1007/s11259-023-10087-0

**Published:** 2023-03-15

**Authors:** Sergio Villanueva-Saz, Marivi Martínez, Pablo Rueda, Sara Bolea, María Dolores Pérez, Maite Verde, Andrés Yzuel, Ramón Hurtado-Guerrero, Julián Pardo, Llipsy Santiago, Antonio Fernández, Maykel Arias

**Affiliations:** 1 Clinical Immunology Laboratory, Veterinary Faculty, University of Zaragoza, 50013 Zaragoza, Spain; 2 Department of Animal Pathology, Veterinary Faculty, University of Zaragoza, Zaragoza, Spain; 3 Instituto Agroalimentario de Aragón-IA2 (Universidad de Zaragoza-CITA), Zaragoza, Spain; 4 Department of Animal Production and Sciences of the Food, Veterinary Faculty, University of Zaragoza, Zaragoza, Spain; 5 Institute for Biocomputation and Physics of Complex Systems (BIFI), University of Zaragoza, Edificio I+D, Campus Rio Ebro, Zaragoza, Spain; 6 Aragon I+D Foundation (ARAID), Zaragoza, Spain; 7 Aragon Health Research Institute (IIS Aragón), Zaragoza, Spain; 8 Department of Microbiology, Pediatrics, Radiology and Public Health, Zaragoza University, Zaragoza, Spain; 9 CIBER de Enfermedades Infecciosas, Instituto de Salud Carlos III, Madrid, Spain; 10 Institute of Carbochemistry, CSIC, Madrid, Spain

**Keywords:** Cat, ELISA, Long-term persistence, SARS-CoV-2, Serology, VNT

## Abstract

Severe Acute Respiratory Syndrome Coronavirus 2 is the causative agent of Coronavirus Disease 2019 in humans. To date, little is known about the persistence of antibodies against SARS-CoV-2 in animals under natural conditions, in particular susceptible pets such as cat. This study reports the detection and monitoring of the humoral response against SARS-CoV-2 including the detection of immunoglobulins G specific for receptor binding domain of SARS-CoV-2 spike protein by an enzyme-linked immunosorbent assay and neutralizing antibodies by virus neutralization assay. Results showed that these antibodies last longer than 16 months in two naturally apparently healthy infected cats with the absence of clinicopathological findings during the follow-up. Moreover, re-infection is also possible with an important increase in virus neutralization test titers in both animals with no evident systemic signs found during each physical examination and with values of hematologic and biochemical parameters inside the normal reference intervals. Our results confirm a slow but progressive decrease of the kinetics and immunity of neutralizing antibodies in cats after the infection. Furthermore, similar to humans SARS-CoV-2 reinfection can stimulate an increase of the neutralizing antibodies determined by these two serological techniques in domestic cats.

## Introduction

SARS-CoV-2 is a coronavirus and the causative agent of the new coronavirus disease 2019 (COVID-19) in humans. This zoonotic betacoronavirus is capable of infecting different animals, being the transmission of SARS-CoV-2 virus from human to domestic animals, the most common route in pets (Kannekens-Jager et al. 2022). Among pets, ferrets, cats and Golden Syrian hamster are the most susceptible animals to SARS-CoV-2 infection (Mahdy et al. 2020; Blaurock et al. 2022).

Nowadays, there are different evidences available showing that animals can be infected and some of these animals develop clinical signs. However, there are insufficient data regarding the level of humoral immunity to SARS-CoV-2 virus and the long-term persistence of antibodies. Some reports determined that humoral immune response is detected during several months in infected pets including dogs (47 days) (Miró et al. 2021), cats (110 days) (Zhang et al. 2020) and ferrets (129 days) (Giner et al. 2021). In contrast long persistence of neutralizing antibodies has only been reported in a case series in pets including seven dogs and two cats (Decaro et al. 2022) detected by four different serological tests. Considering wildlife, there is only a report that confirms that the persistence of SARS-CoV-2 neutralizing antibodies longer than 13 months in naturally infected captive white-tailed deer (*Odocoileus virginianus*) detected by plaque reduction neutralization test (PRNT) (Hamer et al. 2022).

There are numerous serological methods to detect the presence of anti-SARS-CoV-2 antibodies, however, virus neutralization test (VNT) is considered the gold standard technique of humoral response against the virus (Miró et al. 2021). In this sense, the detection of neutralizing antibodies is essential to understanding of immune response by serological methods such as VNT and PRNT or the detection of antibodies against the receptor binding domain (RBD) of SARS-CoV-2 spike protein by ELISA (Santiago et al. 2021). These anti-RBD can disrupt the interaction between receptor binding domain of SARS-CoV-2 spike protein and the mammal angiotensin-converting enzyme 2 protein (ACE2), being considered as neutralizing antibodies too (Barnes et al. 2020).

Our study highlights the immunological observations based on detecting neutralizing antibody longevity in two natural re-infected cats by SARS-CoV-2 together with follow-up data providing information on the humoral response and the absence of clinicopathological alterations.

## Materials and methods

### Case history

The first case was a 2-year-old, indoor spayed female European Shorthair cat with no history of clinical disease or underlying condition. This cat was living in a household with a family composed by three members diagnosed with SARS-CoV-2 infection and clinical signs of COVID-19 All family members had a confirmed diagnosis of COVID-19 by molecular test on January 21, 2021. Sixteen months later, SARS-CoV-2 reinfection occurred on June 24, 2022, one out of three people living in the household had a confirmed diagnosis of COVID-19 by antigen testing.

The second case was a 2.5-year-old, indoor spayed female Persian cat classified as healthy animal with no evident clinical signs. The cat was living in a household with a family composed by four member COVID-19 positive clinical signs confirmed by molecular test on January 8, 2021. Similarly, to the first case, SARS-CoV-2 reinfection of two of four household members with COVID-19 positive result by antigen testing, occurred on June 20, 2022. Both animals were examined at the Veterinary Teaching Hospital Zaragoza University during the follow-up. Cats came to the veterinarian for examination associated to a previous research project (PROGRAMA COVID19 SANTANDER UNIZAR) to detect the presence of seropositive pets.

### Detection of SARS-CoV-2 antibodies (anti-RBD) by in-house ELISA

Antibodies to SARS-CoV-2 were determined by an indirect ELISA for the detection of IgG specific for RBD, described previously (Villanueva-Saz et al. 2022a). The cutoff was set to 0.30 Optical Density units (OD) (mean + 3 standard deviations of values from 92 cats obtained prior the COVID-19 situation in 2015) and the results above this value were considered positive (Saraswati et al. 2019). OD between results obtained during each sampling number during the follow-up were considered different when OD variation was greater than 10%.

### Micro-neutralization assay of SARS-CoV-2

This virus neutralization test (VNT) was performed as described previously (Villanueva-Saz et al. 2022b). The neutralization ID50 was calculated as the highest dilution that protected more than 50% of the cells in the wells from cytopathic effect.

### Laboratory data collection for hematologic and biochemistry analysis

Blood samples were collected from the jugular or cephalic veins into tube with anticoagulant (EDTA) for complete blood count (CBC) using an automatic haematological counter IDEXX Procyte Dx (IDEXX laboratories, Westbrook, ME, USA). In the case of biochemistry profile, blood samples were collected in a plain tube to obtain serum. Serum samples were analysed with an automatic veterinary chemistry analyser (AmiShield, Protect Life International Biomedical Inc., Taiwan) to determine the following parameters: glucose, total protein concentrations, albumin, blood urea nitrogen, creatinine, calcium, inorganic phosphorus, alanine aminotransferase, aspartate aminotransferase, alkaline phosphatase, gamma glutamyl transferase, total bilirubin, amylase, globulins, and serum amyloid A. These samples were stored at 4º C for a maximum of 12 h. Three aliquots from each serum sample were stored at -80 °C until testing. All the samples used in this study to detect the presence of anti-RBD by the in-house ELISA and VNT were thawed only once. Serum protein electrophoresis was also performed by agarose gel electrophoresis system (Hydragel Kit 1–2, Sebia, Evry, France). The electrophoretic curve was displayed and read with Gelscan (Sebia, Evry, France). Laboratory parameters were considered altered when they were outside the reference intervals. Futhermore, the presence of antibodies against the feline coronavirus (FCoV) was tested by a rapid immunochromatographic test *FAST*est® FIP (MEGACOR Diagnostik, Hörbranz, Austria).

## Results

### Case clinical observation and clinicopathological findings

Both case 1 and case 2 were asymptomatic during the follow-up and after the SARS-CoV-2 reinfection. Moreover, none clinicopathological abnormalities were detected by CBC, biochemical profile and serum protein electrophoresis. A negative result was also obtained by the rapid test to detect the presence of antibodies against FCoV in both cases.

### Analysis of serum RBD specific IgG by ELISA and neutralizing antibodies by VNT

The serological results are shown in Table 1. In both cases, the presence of neutralizing antibodies was detected at the first sampling, 40 days later after positive diagnosis of COVID-19 in all family members. In general, case 1 and case 2 had a positive result by ELISA and VNT during the different time points included in the present study. An agreement between results obtained by ELISA and VNT was obtained in all time points, with positive result by both techniques. However, the antibody kinetics seems to be something different considering ELISA or VNT. In the case of ELISA test, a progressive decrease of the OD was observed, being this decrease faster than results obtained by VNT. In this sense, case 1 had an OD slightly higher (0.31) than the detection limit (0.30) one year later but VNT result was stable with no dilution variation (1/320). By contrast, for case 2, one year later, OD (0.72) detected by ELISA was higher than for case 1 with a reduction of VNT value from 1/640 (previous time point) to 1/480.

**Table 1:** Serological follow-up for SARS-CoV-2 antibodies by in-house ELISA and VNT in the cats evaluated

Animal identification	Breed	COVID-19 household positive	Sampling number	Date of collection	RBD ELISA	Follow-up OD variation	VNT (ID50)	Follow-up VNT variation	Anti-SARS-CoV-2 persistence
1	European Shorthair Cat	21-January-2021	1	02-March-2021	0.77		1/640		> 12 months
			2	15-April-2021	0.72	Stable	1/640	Stable	
			3	01-June-2021	0.77	Stable	1/320	Reduced	
			4	29-September-2021	0.78	Stable	1/320	Stable	
			5	08-March-2022	0.31	Reduced	1/320	Stable	
		24-June-2022 (reinfection)	6	19-July-2022	1.36	Increased	1/1920	Increased	> 16 months after SARS-CoV-2 infection
2	Persian Cat	08-January-2021	1	15-March-2021	0.95		1/640	Stable	> 12 months
			2	15-April-2021	1.20	Increased	1/640	Stable	
			3	01-June-2021	1.12	Stable	1/640	Stable	
			4	29-September-2021	1.10	Stable	1/640	Stable	
			5	08-March-2022	0.72	Reduced	1/480	Reduced	
		20-June-2022 (reinfection)	6	13-September-2022	0.87	Increased	1/640	Increased	> 18 months after SARS-CoV-2 infection

ID50: 50% inhibitory dose; OD: Optical density; RBD: receptor binding domain; VNT: virus neutralization test

## Discussion

The evaluation and study of the humoral immune response against SARS-CoV-2 virus based on detecting neutralizing antibodies is essential and of interest to understand immune-mediated mechanisms that might help to protect susceptible animals from SARS-CoV-2 reinfection. To the best of our knowledge, this is the first report that demonstrates that the SARS-CoV-2 reinfection in cats causes an increase of the neutralizing antibodies after the reinfection under natural conditions detected by two different serological methods including ELISA test and VNT in two healthy cats.

Results of antibody longevity after 12 months detected in this study are in accordance with those reported in several longitudinal studies in humans (Yang et al. 2022) and pets including dogs and cats (Decaro et al. 2022).

In human medicine, there is a controversial aspect about the anti-SARS-CoV-2 antibody level and the clinical status of the COVID-19 patients. Some studies detected a more rapid decrease in antibody levels in patients with mild symptoms or asymptomatic compared to patients with more severe clinical signs (Long et al. 2020). By contrast, other study detecting the presence of anti-RBD antibodies found antibodies responses up to 12 months after infection in non-hospitalised people with mild symptoms (Sarjomaa et al. 2022).

In veterinary, there is a lack of information about the antibody persistence except for one study published in Italy with the detection of SARS-CoV-2 neutralizing antibodies in pets up to 10 months since the first positive result using four different serological methods (Decaro et al. 2022). Some differences can be observed between this study and our results, mainly the type of technique performed using two commercial ELISAs focused on detecting nucleocapsid protein antigen. In our study, we have performed an in-house anti-RBD ELISA based on spike protein as antigen and concordant positives results were obtained at all time points, confirming our previous results in humans in which we found that the level of RBD specific IgG correlated with serum viral neutralizing activity (Santiago et al. 2021). However, discordant results among commercial ELISAs and the remaining serological methods to detect neutralizing antibodies were observed in the other study (Decaro et al. 2022). This situation could be explained due to antibody levels against nucleocapsid decline more rapidly than those of anti-RBD antibodies (Lumley et al. 2021; Van Elslande et al. 2021). However, the most likely reason for these differences is that in contrast to anti-RBD antibodies, anti-nucleocapsid antibodies are not expected to present neutralizing activity against the virus and thus its presence would not correlate with VNT results as shown with other viruses like herpes (Papp-Vid et al. 1979). In our study, differences between antibody titers between ELISA and VNT are detecting during the different time points included. One possible explanation is the fact that ELISA technique detects mainly immunoglobulin (Ig) G but there may be other isotypes such as IgA or IgM that can contribute to VNT (Pang et al. 2021).

In addition, no previous information concerning the antibodies kinetics in animals regarding SARS-CoV-2 reinfection have been published. Our results showed that the level of antibodies decreased with time after infection, as detected by ELISA at different time points. In these cats, the presence of a new reinfection stimulated the antibody response detected by an increase in the production of neutralizing antibodies. In conclusion, our study demonstrates the persistence of neutralizing antibodies up 12 months after the first sampling in the cats evaluated. Moreover, SARS-CoV-2 reinfection promotes an increase of the immune response with an elevation of antibody titers detected by anti-RBD ELISA and VNT.

## Data Availability

The data that support the findings of this study are available from the corresponding author upon reasonable request.
